# Exploring the response of yellow lupine (*Lupinus luteus* L.) root to drought mediated by pathways related to phytohormones, lipid, and redox homeostasis

**DOI:** 10.1186/s12870-024-05748-4

**Published:** 2024-11-06

**Authors:** Sebastian Burchardt, Małgorzata Czernicka, Agata Kućko, Wojciech Pokora, Małgorzata Kapusta, Krzysztof Domagalski, Katarzyna Jasieniecka-Gazarkiewicz, Jacek Karwaszewski, Emilia Wilmowicz

**Affiliations:** 1 Chair of Plant Physiology and Biotechnology, Faculty of Biological and Veterinary Sciences, Nicolaus Copernicus University, 1 Lwowska Street, Toruń, 87-100 Poland; 2 Department of Plant Biology and Biotechnology, Faculty of Biotechnology and Horticulture, University of Agriculture in Krakow, Al. Mickiewicza 21, Krakow, 31-120 Poland; 3 Department of Plant Physiology, Institute of Biology, Warsaw University of Life Sciences- SGGW, Nowoursynowska 159, Warsaw, 02-776 Poland; 4 Department of Plant Physiology and Biotechnology, University of Gdańsk, 59 Wita Stwosza, Gdańsk, 80-308 Poland; 5 Bioimaging Laboratory, University of Gdańsk, 59 Wita Stwosza, Gdańsk, 80-308 Poland; 6 Department of Immunology, Faculty of Biological and Veterinary Sciences, Nicolaus Copernicus University, 1 Lwowska Street, Toruń, 87-100 Poland; 7 Intercollegiate Faculty of Biotechnology, University of Gdansk and Medical University of Gdańsk, Abrahama 58, Gdańsk, 80-307 Poland

**Keywords:** Lipoxygenase, NGS, Phospholipase D, Phytohormones, Redox balance, RNA-seq, Yellow lupine

## Abstract

**Background:**

Yellow lupine (*Lupinus luteus* L.) is a high-protein crop of considerable economic and ecological significance. It has the ability to fix atmospheric nitrogen in symbiosis with *Rhizobium*, enriching marginal soils with this essential nutrient and reducing the need for artificial fertilizers. Additionally, lupine produces seeds with a high protein content, making it valuable for animal feed production. However, drought negatively affects lupine development, its mutualistic relationship with bacteria, and overall yield. To understand how lupine responds to this stress, global transcriptome sequencing was conducted, along with in-depth biochemical, chromatography, and microscopy analyses of roots subjected to drought. The results presented here contribute to strategies aimed at mitigating the effects of water deficit on lupine growth and development.

**Results:**

Based on RNA-seq, drought-specific genes were identified and annotated to biological pathways involved in phytohormone biosynthesis/signaling, lipid metabolism, and redox homeostasis. Our findings indicate that drought-induced disruption of redox balance characterized by the upregulation of reactive oxygen species (ROS) scavenging enzymes, coincided with the accumulation of lipid-metabolizing enzymes, such as phospholipase D (PLD) and lipoxygenase (LOX). This disruption also led to modifications in lipid homeostasis, including increased levels of triacylglycerols (TAG) and free fatty acids (FFA), along with a decrease in polar lipid content. Additionally, the stress response involved alterations in the transcriptional regulation of the linolenic acid metabolism network, resulting in changes in the composition of fatty acids containing 18 carbons.

**Conclusion:**

The first comprehensive global transcriptomic profiles of lupine roots, combined with the identification of key stress-responsive molecules, represent a significant advancement in understanding lupine’s responses to abiotic stress. The increased expression of the *Δ12DESATURASE* gene and enhanced PLD activity lead to higher level of linoleic acid (18:2), which is subsequently oxidized by LOX, resulting in membrane damage and malondialdehyde (MDA) accumulation. Oxidative stress elevates the activities of superoxide dismutase (SOD), ascorbate peroxidase (APX), and catalase (CAT), while the conversion of FFAs into TAGs provides protection against ROS. This research offers valuable molecular and biochemical candidates with significant potential to enhance drought tolerance . It enables innovative strategies in lupine breeding and crop improvement to address critical agricultural challenges.

**Supplementary Information:**

The online version contains supplementary material available at 10.1186/s12870-024-05748-4.

## Background

Drought significantly impairs the formation and functioning of plant roots, which is particularly detrimental for yellow lupine (*Lupinus luteus* L.) due to its symbiotic relationship with soil bacteria of the genus *Rhizobium* [1]. These bacteria fix atmospheric nitrogen (N_2_) into ammonia (NH_3_), thereby enriching nutrient-poor soils with nitrogen, a critical element for subsequent plant development [2–4]. Consequently, it reduces the need for nitrogen fertilizers and additional agrotechnical interventions. Drought-induced disruptions in root function severely impact lupine yield, primarily through increased flower abortion [5–7]. The unpredictability of yield poses a significant challenge for the agricultural sector. Perception of the drought stimuli by sensors – roots [8], initiates a signaling cascade involving phytohormones, such as abscisic acid (ABA), and ethylene (ET), along with reactive oxygen species (ROS) - primary mediators of stress signaling [7]. Among ROS, hydrogen peroxide (H_2_O_2_) is particularly important, as it plays a key role in cell wall remodeling, a process essential for *Rhizobium* penetration [9]. However, excessive production of ROS can lead to oxidative stress, necessitating the activation of protective antioxidant systems such as superoxide dismutase (SOD), catalase (CAT), and peroxidase (POX), which function in a tightly coordinated manner. Specifically, the superoxide radicals produced by SOD are converted to H_2_O_2_, which then serves as substrate for POX and CAT facilitating the detoxification of ROS [10, 11]. Additionally, ROS promote the release of polyunsaturated fatty acids (PUFAs) from cellular membranes, thereby increasing membrane permeability [7, 12, 13], a critical feature for stress responses. This release is mediated by lipase (LIP) activity, resulting in the production of linoleic and/or α-linolenic acids – substrates for lipoxygenase (LOX) [14]. Both these reactions are essential for the biosynthesis of jasmonates (JAs), stress-responsive phytohormones [15] that play a vital role in root system morphogenesis and function. For example, exogenous JAs (jasmonic acid, JA and methyl jasmonate, MeJA) activate *NOD* genes in *Rhizobium leguminosarum* and *Bradyrhizobium japonicum*, consequently initiating nodulation and enhancing nitrogen fixation in *Glycine max* L. and *Phaseolus vulgaris* L [16–18].

As we have previously shown, drought stress reduces the number of nodules in *L. luteus*, leading to progressive degradation of symbiosomes [4]. This degradation is associated with decreased leghemoglobin content, which is essential for facilitating oxygen supply to nitrogen-fixing bacteria [19]. Furthermore, drought stress activates numerous genes and proteins involved in regulatory pathways that maintain the homeostasis of stress phytohormones and reorganize cell wall structure [4, 19]. Up to date, no paper documents the global transcriptomic profile of *L. luteus* roots under drought conditions. That is why drought-responsive genes were identified and categorized into various regulatory pathways, including those related to phytohormones, lipid metabolism, and redox homeostasis. Investigations were directed toward these pathways, and additional biochemical, microscopy, and instrumental analyses were conducted. Our research provides novel insight into the genetic and biochemical components activated by drought in roots. This serves a basis for the understanding of the crop-specific molecular responses to drought stress.

## Methods

### Plant material and growth conditions

The plant material used in this study was a commercial wild-type cultivar of yellow lupine (*Lupinus luteus* L.) – ‘Taper’. The seeds were provided by Poznańska Hodowla Roślin Ltd., Tulce, Poland. The plants were grown in phytotron chambers under controlled conditions as optimized and described previously by Frankowski et al. [20]. Throughout the 34-day cultivation period, plants were watered with an amount of water appropriate to their developmental stage (the same for each pot). Subsequently, half of the plants underwent a 14-day drought period at 25% water holding capacity (WHC) according to the protocol of Wilmowicz et al. [7]. The remaining lupines served as a control group under optimal moisture conditions (70% WHC). Lupine root is composed of 2 parts: the upper part, which includes nodules, and the lower part without nodules. For our experiments, the upper part of the root collected on the 48^th^ day of plant growth was used. Nodules were excised, and the root tissue was used for transcriptome analysis, lipid profiling, Western blot, and malondialdehyde (MDA) assays. Roots with nodules intact were collected separately for microscopy and ROS measurements. For the microscopy analyses, the material was immediately fixed, while tissues for other experiments were frozen in liquid nitrogen. Collected material for RNA-seq and biochemical analyses was stored at -80℃.

### RNA-seq library construction and Illumina NovaSeq6000 sequencing

Root fragments were ground in liquid nitrogen using a mortar and pestle. Total RNA extraction was carried out with the NucleoSpin^®^ RNA Plant and Fungi kit (Macherey-Nagel Inc, Pennsylvania USA) following the manufacturer’s instructions, which included DNAse treatment. RNA concentration and purity were assessed fluorometrically using the Qubit™ RNA BR Assay Kit (Invitrogen™, Waltham, Massachusetts, USA). RNA integrity numbers (RINs) of each biological repetition were evaluated using the 2100 Bioanalyzer System (Agilent 2100 Bioanalyzer; Agilent Technologies, Palo Alto, Santa Clara, CA, USA). Four cDNA libraries were constructed using the NEBNext^®^ Ultra™ RNA Library Kit (Illumina, San Diego, CA, USA). High-throughput RNA sequencing was performed in PE150 (paired ends mode, with 150 bp read length) using an Illumina NovaSeq6000 (Illumina, San Diego, CA, USA). All RNA-seq datasets generated in this study have been deposited in the NCBI SRA database under BioProject PRJNA890161. SRA numbers for control and drought samples are SRX17888545 and SRX17888546, respectively.

### Bioinformatic analysis of RNA-seq data

The raw RNA-seq reads underwent initial quality assessment using FastQC v0.11.5 (http://www.bioinformatics.bbsrc.ac.uk/projects/fastqc/) and were filtered using Trimmomatic v0.39 (https://github.com/usadellab/Trimmomatic, Bolger et al. 2014). The quality filter was applied with the following parameters: a Phred score (Q) = 20, minimal read length = 25 bp, and exclusion of unpaired reads. After quality control, the cleaned reads were used for *de novo* assembly performed using the Trinity v2.4.0 (https://github.com/trinityrnaseq/trinityrnaseq/wiki). All unigenes and transcripts were annotated using Trinotate v3.2.2 (https://github.com/Trinotate/Trinotate.github.io/wiki). Protein-coding regions were identified through BLASTX and BLASTP by searching the UniProt database. Identification of functional protein domain (PFAM / Hmmer), Gene Ontology (GO), orthology relationship (EggNOG), and Kyoto Encyclopedia of Genes and Genomes (KEGG) database (http://www.genome.jp/kegg/) were determined. The number of reads mapped to unigenes and transcripts in each library was quantified with RSEM v1.3.3 (https://github.com/deweylab/RSEM) software. Read counts for individual transcripts and unigenes were obtained from the mapping results and normalized to facilitate reliable expression comparisons between samples by specifying the number of fragments per kilobase of exon per million mapped fragments (FPKM). Differentially expressed genes (DEGs) were calculated using the Empirical Analysis of Digital Gene Expression-edgeR statistical package, while the trimmed mean of M-values (TMM) method was used to calculate normalization factors. Transcripts with a False Discovery Rate (FDR) < 0.05 were considered as significantly differentially expressed. GO enrichment analysis was conducted with topGO in R/Bioconductor package v2.34.0 [21] using classic and elim methods with Fisher’s test (*p* < 0.01). KEGG pathway enrichment of DEGs was carried out using clusterProfiler v3.16.1 [22]. Due to the unavailability of NCBI Entrez identifiers for *L. luteus*, DEGs were mapped to KEGG orthologs and pathways using KofamKOALA (https://www.genome.jp/tools/kofamkoala)] with an E-value cut-off of 0.01.

### Lipid profiling

Total lipids were extracted according to a modified method described by Bligh and Dyer [23]. Plant tissues were homogenized in a mixture of 3.75 mL of chloroform: methanol (1:2, v/v) and 1.25 mL of 0.15 M acetic acid. In the next step, 1.25 mL of chloroform and 1.25 mL of distilled H_2_O were added to the homogenate. The obtained extract was transferred to glass tubes and centrifuged (2000 rpm) for 2 min at 4ºC. The chloroform phase containing the lipids was transferred to fresh glass test tubes, dried under a stream of N_2_ at 30ºC, and finally reconstituted in 2 mL chloroform. After evaporation of the chloroform, aliquots of these lipid extracts were methylated with a mixture of 2% sulfuric acid (H_2_SO_4_) in dry methanol at 90ºC for 1 h. Then 50 nmol of methyl heptadecanoate (17:0-Me) was added to the mixtures as an internal standard. The resulting fatty acid (FAs) methyl esters were extracted with n-hexane and analyzed by a gas chromatograph (GC-2010; Shimadzu, Kyoto, Japan) equipped with a 60 m × 0.25 m CP-WAX 58-CB column (Agilent Technologies, Santa Clara, CA, USA) and a flame-ionization detector. The lipid classes profile of chloroform extracts (prepared as described above) was determined by thin-layer chromatography on silica gel plates (Supelco Inc, Merck, Kenilworth, NJ, USA) with a solvent system of hexane: diethyl ether: acetic acid (70:30:1, v/v/v). Lipid classes were visualized using a 2% solution of primuline in acetone, and the plate was then exposed to UV light. Silica gel corresponding to each lipid class was scraped from the marked areas on the plate. These lipids were methylated in situ and subsequently analyzed by gas chromatography as described earlier.

### Western blot analysis

Frozen plant material (~ 0.4 g) was powdered in liquid nitrogen using a mortar and pestle and homogenized in 1 ml of extraction buffer containing 50 mM Tris-HCl, pH 8.0; 300 mM NaCl; 10% v/v glycerol; and 1 mM ethylenediaminetetraacetic acid tetrasodium salt dihydrate (EDTA). The homogenates were then centrifuged (4 °C, 10 min) and the protein-containing supernatants were collected for further analyses. Total protein concentration was determined according to the Bradford method [24]. Denatured proteins (25 µg) were separated by electrophoresis (sodium dodecyl-sulfate polyacrylamide gel electrophoresis, SDS-PAGE) on 4–12% (w/v) polyacrylamide gels in a Criterion™ Cell apparatus (Bio-Rad, Hercules, USA). Subsequently, proteins were transferred onto a nitrocellulose membrane, which was blocked with 1% bovine serum albumin (BSA) solution in tris-buffered saline (TBS) buffer (pH 7.5) for 1 h before electrotransfer. Simultaneously, the second gel was stained with Coomassie Brilliant Blue. For immunodetection of PLD and LOX, the nitrocellulose membrane was incubated at 4 °C for 12 h with either anti-PLD antibody (AS09 556) or anti-LOX antibody (AS06 128), each diluted 1:1000 in a 0,5% BSA solution prepared in TBS buffer pH 7.5. Then, the membrane was washed 3 times with TBS buffer pH 7.5 and incubated for 2 h at room temperature with DyLight 488-conjugated anti-rabbit IgG (AS09 633), diluted 1:10000 in TBS buffer pH 7.5. All antibodies were provided by Agrisera (Sweden). Subsequently, proteins recognized by the antibodies were detected with an ECL SuperBright (AS16 ECL-SN, Agrisera, Sweden), and the signal was captured using in a ChemiDoc™ Touch Imaging System. Densitometry analysis of the immunoblots was performed using ImageJ software, and average densitometry values were presented on charts.

### Tissue fixation and immunofluorescence experiments

Fresh material for microscopy analyses was excised and prefixed in 3% (v/v) N-(3-dimethylaminopropyl)-N’-ethylcarbodiimide hydrochloride (EDAC) solution for 12 h at 4 °C. Subsequently, samples were processed following our standard protocol involving dehydration, supersaturation, and embedding [25]. Section (1 μm thickness) were cut using an Ultracut microtome (Reichert-Jung, Germany), mounted on glass slides, and subjected to the reactions with PLD (AS09 556), and LOX (AS06 128) antibodies provided by Agrisera (Vännäs, Sweden). Antibodies were diluted 1:50 in BSA dissolved in PBS pH 7.2. For immunolabeling, sections were incubated (4 °C) overnight with the primary antibodies, followed by three washes in phosphate buffered saline (PBS, pH 7.2) for 10 min. each. Then secondary antibodies (anti-rabbit DyLight IgG Alexa Fluor 488-conjugated IgG, AS09 633, Agrisera, Vännäs, Sweden) were served for 2 h at 37 °C. Observations and documentation of results were carried out using a confocal microscope (Leica Stellaris 5 WLL, Leica, Wetzlar, Germany).

### Determination of H_2_O_2_ and MDA content

The concentration of H_2_O_2_ was determined following the method previously described [7]. In turn, the protocol outlined by Hodges et al. [26] was followed for MDA analysis, using thiobarbituric acid (TBA) with modifications detailed in our previous paper [6].

### Enzyme activity assays

Protein extracts were obtained from frozen roots, and SOD activity was assessed according to the indirect method described by Pokora et al. [27] and Tukaj and Pokora [28]. In brief, nondenaturing PAGE was conducted using riboflavin for gel polymerization. Samples containing 50 µg of the protein were then loaded onto the gel. SOD isoforms were visualized by staining with nitro blue tetrazolium chloride (NBT). The gels were exposed to light until a blue coloration developed, with SOD activity indicated by achromatic regions. Isoforms of SOD were differentiated based on their varying sensitivities to H_2_O_2_ and potassium cyanide (KCN). The activity of each SOD isoform was quantified by comparing the relative intensity (mm^2^) of the bands to a copper/zinc superoxide dismutase (Cu/Zn-SOD) reference standard, which has a defined activity of 4.4 U mg^− 1^ protein (Sigma-Aldrich, St. Louis, MO, USA). Electrophoresis, gel documentation, and densitometric analysis were performed using Bio-Rad’s MidiProtean Electrophoresis System, ChemiDoc Imaging System, and Quantity1 software, respectively. In turn, the APX activity was determined spectrophotometrically following the method of Nakano and Asada [29], with slight modifications as described by Aksmann et al. [30]. It is based on the oxidation rate of pyrogallol. Quantity1D software (Bio-Rad, Hercules, California, USA) was used for the densitometric analysis. CAT activity was measured spectrophotometrically according to Aksmann et al. [30], and the results were expressed as the amount of H_2_O_2_ detoxified by CAT.

### Statistical analysis

For the RNA-seq experiment, two biological replicates were prepared for each experimental variant. Each biological replicate was a pool of mixed in equal proportions of RNAs obtained from three independent isolations of material, sourced from at least three plants. Other analyses were conducted with three biological and three technical replicates. Data obtained from these experiments were analyzed using the t-test with significance levels set at *p* ≤ 0.01 or 0.05. Statistical analyses were performed using SigmaPlot. Results are presented as the mean ± SD.

## Results

### RNA-sequencing, *de novo* transcriptome assembly and DEGs identification

To assess transcriptomic changes in *L. luteus* roots under drought stress, RNA-seq analysis was conducted for samples from both control and drought-treated plants. RNA sequencing with the Illumina NovaSeq6000 produced in total 80,441,550 paired-end 150 bp reads corresponding to more than 11.4 billion base pairs. Of these, 41,777,419 reads (52%) were originated from the control sample and 38,664,131 reads (48%) derived from drought-treated samples (Supplementary file 1S1). Pearson correlation analysis between biological replicates was greater than 0.8, indicating the reliability of the RNA-seq results (Supplementary file 1S2).

**Fig. 1:**
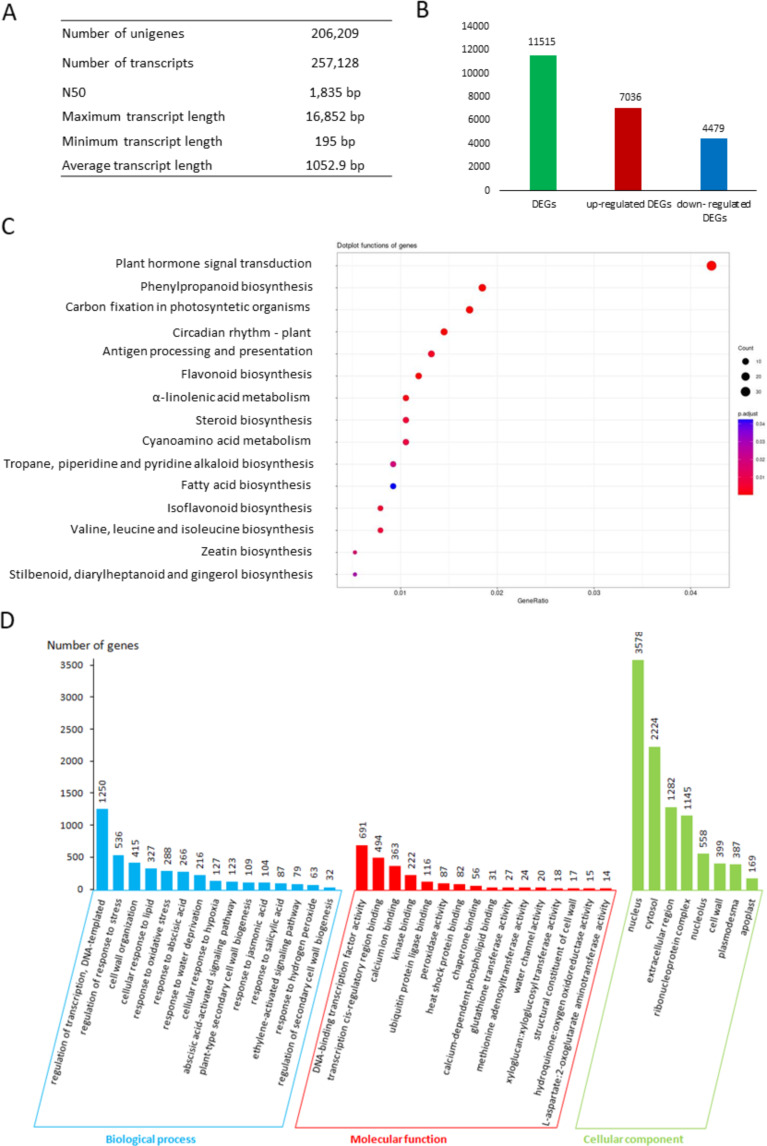
Summary of *de novo* transcriptome assembly of *Lupinus luteus* L. (**A**). Numerical summary of the assembled transcriptome (**B**). Numbers of differentially expressed genes (DEGs) in *L. luteus* roots under drought (FDR < 0.05) (**C**). DEGs enrichment is based on the Kyoto Encyclopedia of Genes and Genomes (KEGG) pathway. The number of core genes (“count”) divided by the total number of genes is the gene ratio. The sizes of the dots represent the number of core genes, and the color indicates the adjusted P-value. Only pathways with p-values < 0.05 were eligible for enriched biological processes (**D**). List of top Gene Ontology (GO) terms enriched by DEGs

The *de novo* transcriptome assembly resulted in 257,128 transcripts corresponding to 206,209 unigenes, with lengths ranging from 195 to 16,852 bp and an average length of 1,053 bp (Fig. 1A). The size distribution of transcripts showed the highest abundance in the 0–500 bp size range (Supplementary file 1S3). Functional annotation of the assembled transcripts involved BLASTx and BLASTp searches against the UniProtKB/SwissProt database, revealing alignments for 176,818 (86%) nucleotide sequences and 98,764 (48%) protein sequences (Supplementary file 1S4A). Searching against the Pfam database identified 72,136 (35%) putative domains in the *L. luteus* transcriptome (Supplementary file 1S4A). GO and KEGG pathway analysis provided functional annotations for 172,177 (83%) and 158,711 (77%) transcripts, respectively (Supplementary file 1S4A). Among all annotated transcripts, over 41.2% showed significant sequence similarity to *Arabidopsis thaliana*, while 58.8% exhibited similarity to other plant species, including 0.7% to legume plants (Supplementary file 1S4B).

A total of 87% of the reads were mapped to the *de novo* assembled transcriptome using the Botwie2 program. Under drought stress conditions, 11,515 genes were differently expressed compared to untreated plants, with approximately 61% (7,036) of the genes showing over-expression and 39% (4,479) of the genes characterized by down-regulated expression (Fig. 1B, Supplementary file 1S5). To identify the biological pathways activated in roots under drought stress, the DEGs were mapped to reference canonical pathways in the KEGG database. The DEGs were significantly enriched in 15 pathways, including plant hormone signal transduction, phenylpropanoid biosynthesis, carbon fixation, circadian rhythm, antigen processing and presentation, flavonoid biosynthesis, α-linolenic acid metabolism, steroid biosynthesis, cyanomino acid metabolism, tropane, piperidine alkaloid biosynthesis, FAs biosynthesis, isoflavonoid biosynthesis, valine, leucine and isoleucine biosynthesis, zeatin biosynthesis and stilbenoid, diarylheptanoid, and gingerol (Fig. 1C, Supplementary file 1S6A). The phytohormone signal transduction category was the most represented by DEGs.

The identified DEGs were also enriched using GO terms. A total of 102 biological processes (BP), 56 molecular functions (MF), and 39 cellular components (CC) terms were found to be significantly enriched in the DEGs (Supplementary file 1S6B). Among the top enriched BP terms were regulation of transcription, DNA-templated, regulation of response to stress, water deprivation, oxidative stress, hydrogen peroxide, as well as a cellular response to lipid, and cell wall organization (Fig. 1D). In the MF category, the most relevant terms were i.e. DNA-binding transcription factor activity, kinase binding, peroxidise activity, heat shock protein binding, calcium-dependent phospholipid binding, structural constituent of the cell wall (Fig. 1D). Regarding the CC terms, the most represented groups among the DEGs were related to the nucleus, cytosol, extracellular region, ribonucleoprotein complex, cell wall, plasmodesma and apoplast (Fig. 1D).

### Hormonal response under drought stress

**Fig. 2:**
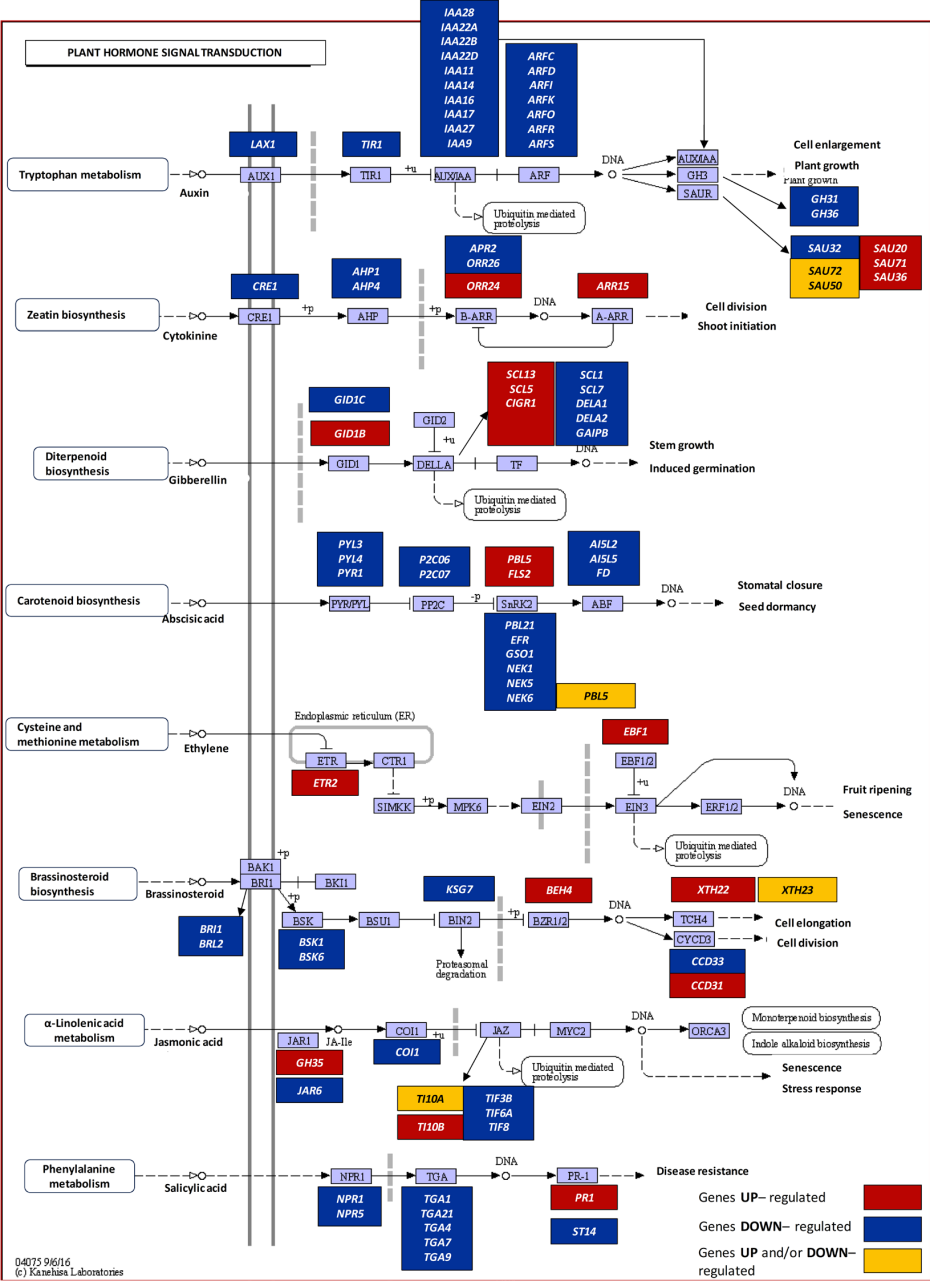
DEG-enrichment of the plant hormone signal transduction pathway (ath04075) conducted using KEGG annotation. Key regulatory components involved in multiple hormone response pathways are highlighted by color (red, up-regulated; blue, down-regulated; yellow, up and/or down-regulated). Transcript IDs and fold changes in transcript abundance are provided in Supplementary file 1S7

In yellow lupine root, drought stress alters the expression of genes involved in both indole-3-acetic acid (IAA) biosynthesis and its signal transduction pathway (Fig. 2, Supplementary file 1S7). A decrease in the expression of genes related to the metabolism of tryptophan (Trp), an indole amino acid formed in chloroplasts that can be converted to IAA, was observed [31]. The other DEGs captured are associated with auxin transport (*AUXIN TRANSPORTER 1*,* LAX1*) and perception (*AUXIN TRANSPORT INHIBITOR RESPONSE*,* TIR*). TIR1 binds auxin and interacts with proteins that act as repressors of transcription factors responsible for regulating the expression of auxin-responsive genes [32]. Moreover, under drought conditions, downregulation of genes encoding auxin early-response proteins containing the auxin responsive (AuxRE) element in their promoter sequences was found. These genes can be categorized into three homologous classes: *AUX/IAA* (*IAA28*,* IAA22A*,* IAA22B*,* IAA22D*,* IAA11*,* IAA14*,* IAA16*,* IAA17*,* IAA27*,* IAA9*), *SMALL AUXIN UP-REGULATED RNA*, *SAUR* (*SAU32I*), and *GRETCHEN HAGEN3*, *GH3* (*GH31*,* GH36*) (Fig. 2, Supplementary file 1S7). In addition, several *SAUR* genes exhibited varied regulation patterns: *SAU20*,* SAU36*, and *SAU71* were upregulated, while *SAU50* and *SAU72* showed both upregulation and downregulation. Our study revealed also a negative impact of drought on the expression of *AUXIN RESPONSE FACTORS*, *ARF* genes (*ARFC*,* ARFD*,* ARFI*,* ARFK*,* ARFO*,* ARFR*,* ARFS*) (Fig. 2, Supplementary file 1S7), encoding auxin response transcription factors that bind to AuxRE in promoter regions [33].

Another plant hormone involved in drought responses in lupine is adenine-derived zeatin (Fig. 2, Supplementary file 1S7), the first identified natural cytokinin (CK) to be identified [34]. The stress resulted in the downregulation of *CYTOKININ RESPONSE 1* (*CRE1*), which encodes the zeatin receptor, as well as *HISTIDINE-CONTAINING PHOSPHOTRANSMITTER1* (*AHP1* and *AHP4*), encoding phosphotransfer protein involved in CK signal transduction from the cytosol to the nuclei [35]. The transcriptional activity of genes encoding type-B ARR family proteins, which modulate early CK responses [36] was also variably regulated by drought. Among them, *5’ADENYLYLPHOSPHOSULFATE REDUCTASE 2* (*APR2*) and *TWO-COMPONENT RESPONSE REGULATOR* (*ORR26*) were downregulated, while *ORR24* was positively influenced by water deficit. Gene encoding the type-A ARR negative regulator of CK signaling (*ARR15*) was also upregulated under stress conditions.

Drought stress significantly influences the biosynthesis pathway of diterpenoids, including gibberellins (GAs) (Fig. 2, Supplementary file 1S7). Two transcripts encoding the GAs receptors, *GID1B* and *GID1C*, were identified, with *GID1B* being upregulated and *GID1C* downregulated, respectively. The binding of hormones to the GID1 receptor promotes interactions with DELLA proteins, the key repressors of the GAs pathway [37]. Our experiments showed substantial drought-induced changes in the transcriptional activity of eight *DELLA* genes. Five of them (*SCL1*,* SCL7*,* DELA1*,* DELA2*,* GAIPB*) were downregulated, while three others (*SCL13*,* SCL5*,* CIGR1*) showed upregulation.

Plant adaptation to adverse conditions is governed by a stress molecule – ABA, a derivative of carotenoids [38]. ABA is perceived by several types of receptors, most notably PYRABACTIN RESISTANCE1 (PYR1)/PYR1-LIKE (PYL)/REGULATORY COMPONENTS OF ABA RECEPTORS (RCAR) [39]. It was shown that drought in lupine led to the downregulation of *PYL3*, *PYL4*, and *PYR1* (Fig. 2, Supplementary file 1S7). In turn, the transcripts of SERINE/THREONINE-PROTEIN KINASES (SnRK2) kinases, which act on downstream components of the ABA-signaling pathway to activate ABA-responsive genes [40], showed differential expression. Specifically, they were downregulated (*PBL21*,* EFR*,* GSO1*,* NEK1*,* NEK5*,* NEK6*), upregulated (*PBL5*,* FLS2*), and exhibited variable regulation, being either up- and downregulated (*PBL5I*). The ABA signaling pathway involves basic-leucine zipper (bZIP) transcription factors transcription factors known as ABA-responsive element binding factors (ABFs) [41]. In lupine roots, drought stress impacted these factors (*AI5L2*,* AI5L5*,* FD*). SnRK2 are counteracted by type 2 C protein phosphatases (PP2Cs), which inhibit the activation of ABA response [42]. Under drought conditions, the expression of two PP2C genes (*P2C06* and *P2C07*) in lupine was downregulated (Fig. 2, Supplementary file 1S7).

ET, another stress hormone, is synthesized via a pathway involving methionine [43]. Under drought stress, the transcriptional activity of *ETHYLENE RECEPTOR2* (*ETR2*) (Fig. 2, Supplementary file 1S7) encoding ET receptor located in the endoplasmic reticulum, as well as expression of *ETHYLENE INSENSITIVITY 3-BINDING F-BOX PROTEIN* (*EBF1*) (Fig. 2, Supplementary file 1S7) encoding nuclear-localized component of Cullin 1-based E3 complexes repressing ET signaling by *ETHYLENE INSENSITIVITY 3* (*EIN3*) degradation, was were also increased [44].

In addition, genes related to the brassionosteroid (BR) signaling were annotated in the lupine root transcriptome (Fig. 2, Supplementary file 1S7). Among those negatively regulated by drought genes were encoding membrane-localized BRI1 (*BRASSINOSTEROID INSENSITIVE*, *BRI1*, and *BRI2*) and BR-signaling cytoplasmic receptor-like kinases (*BSK1* and *BSK6*), as well as the negative BR signaling regulator (*BRASSINOSTEROID INSENSITIVE PROTEIN 2*, *KS7G*) [45]. Conversely, *BEH4* (*BRASSINOSTEROID RESISTANT 1/2* gene) (Fig. 2, Supplementary file 1S7) encoding the key transcription factor of BR signaling, was upregulated. Drought stimulated the transcriptional activity of *XYLOGLUCAN: XYLOGLUCOSYL TRANSFERASES* (*XTH22* and *XTH23*), although *XTH22* also showed downregulation under stress. XTH enzymes, members of the xyloglucan endotransglucosylases family, respond to BRs due to specific promoter fragments [46]. Drought differentially regulated the expression of BR-responsive cyclin D3 genes involved in cell division: *CCD31* (*CYCLIN* D3) was upregulated, while *CCD33* was negatively influenced by stress.

Drought in lupine negatively affected components related to salicylic acid (SA) biosynthesis from phenylalanine (Fig. 2, Supplementary file 1S7). This included the downregulation of key signaling components; *NONEXPRESSOR OF PATHOGENESIS-RELATED* gene (*NPR1*) and *NPR5* coding SA receptors, and several *TGA* genes (*TGACG-BINDING FACTOR*) (*TGA1*, *TGA4*, *TGA7*, *TGA9*, *TGA21*) encoding transcription factors regulating SA-induced *PATHOGENESIS-RELATED* (*PR*) gene expression [47]. *NPR1* essential for SA-mediated *PRs* gene expression, were up- (*PR1*) and downregulated (*ST14*) under water deficit in lupine.

In lupine roots, drought stress influenced the signaling of lipid-derived jasmonates (JAs) at the transcriptional level (Fig. 2). Free JA could be modified through enzymatic processes involving proteins such as JAR (JASMONATE RESISTANT1) and GH3, which converts JA to the active form, jasmonoyl-isoleucine (JA-Ile) [48]. Our study revealed distinct expression patterns of *JAR6* and *GH35* genes, with *JAR6* downregulated and *GH35* upregulated (Fig. 2, Supplementary file 1S7). Similar trends were observed for genes encoding repressors of JAs signaling – jasmonate ZIM domain-containing proteins (JAZs); *TIF3B*,* TIF6A*, and *TIF8* were downregulated, *TI10B* was upregulated, while *TI10A* was up- and/or downregulated (Fig. 2, Supplementary file 1S7). Furthermore, drought suppressed the transcriptional activity of *COI1* (Fig. 2, Supplementary file 1S7), which encodes CORONATINE-INSENSITIVE PROTEIN1 – JA-Ile receptor.

### Drought-evoked modifications of lipid metabolism

**Fig. 3:**
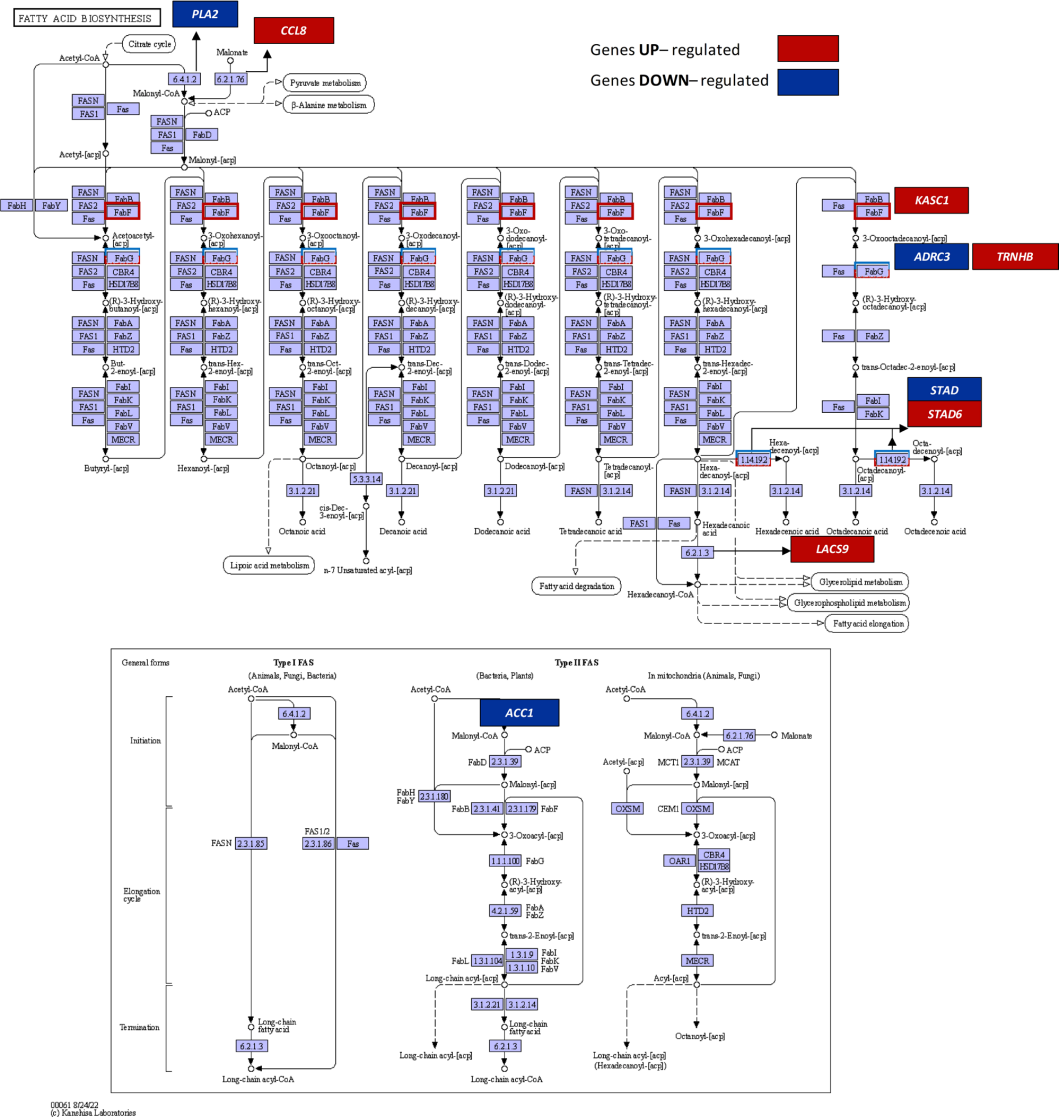
KEGG pathway of fatty acid metabolism (map00071). The marked DEGs in red and blue boxes were up- and downregulated in lupine roots under drought, respectively. Transcript IDs and fold changes in transcript abundance can be found in Supplementary file 1S7

Drought significantly altered the transcriptional activity of enzymes involved in lipid metabolism in lupine roots (Fig. 3, Supplementary file 1S7). Our study identified 7 genes related to FAs biosynthesis (Fig. 3, Supplementary file 1S7). Two genes i.e. *ACETYL-COA CARBOXYLASE* (*ACC1*) and *ACETYL-COA CARBOXYLASE/BIOTIN CARBOXYLASE 1* (*ACACA*), which catalyze the initial step of FAs synthesis by converting acetyl-CoA to malonyl-CoA, were downregulated under drought conditions. The expression of *3-OXOACYL-[ACYL-CARRIER PROTEIN] REDUCTASE* (*ADRC*), encoding oxidoreductase involved in the first reduction step in FAs biosynthesis, and *ACYL-[ACYL-CARRIER-PROTEIN] DESATURASES* (*STAD* and *STAD6*) responsible for introducing double bond into acyl-ACP substrates, was also influenced positively or negatively by the stress. In turn, upregulation of *KASC1* gene encoding 3-oxoacyl-[acyl-carrier-protein] synthase II, responsible for condensation reaction specific to C-16 elongation to C-18 FA, was observed. Additionally, *LACS*, encoding long-chain acyl-CoA synthetase involved in the activation of long-chain and very-long-chain FAs to form acyl-CoA, was upregulated.

**Fig. 4:**
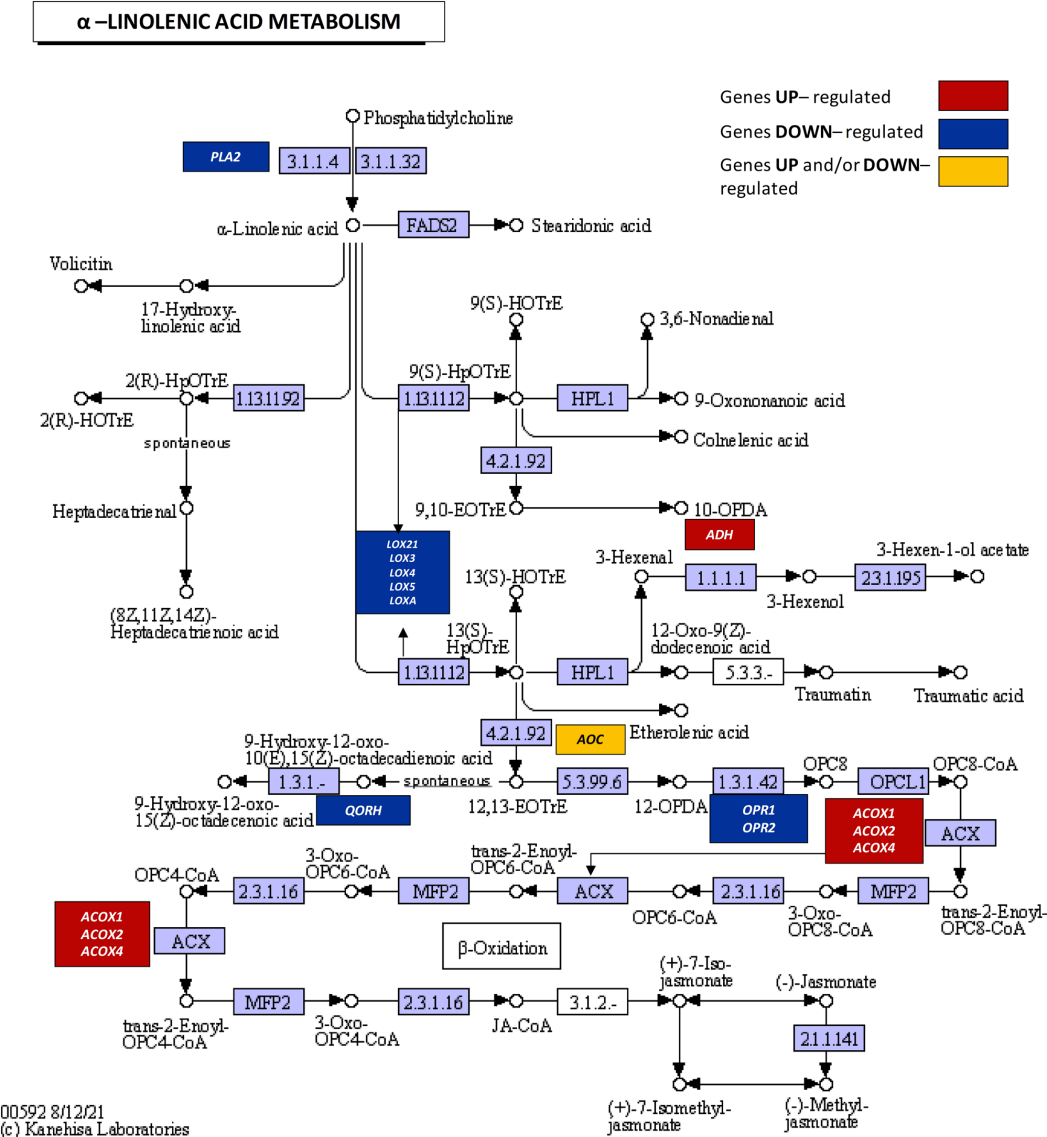
KEGG pathway of α-linolenic acid metabolism (ko00592) highlighting differentially expressed genes (DEGs) identified in lupine roots under drought. Upregulated DEGs are marked with red boxes, downregulated with blue boxes, and genes showing both up- and downregulation are indicated with yellow boxes. Transcript IDs and fold changes in transcript abundance can be found in Supplementary file 1S7

Based on RNA-seq results, DEGs directly involved in *α*-linolenic acid metabolism were identified (Fig. 4, Supplementary file 1S7), which leads to JAs formation. We observed downregulation of *PLA2* encoding phospholipase A, *LOXA*,* LOX3*,* LOX4*,* LOX5*, and *LOX21* encoding lipoxygenases, and *OPR1* and *OPR2* encoding 12-oxophytodienoic acid (OPDA) reductases. Concurrently, there was upregulation of *ACOX1*, *ACOX2*, and *ACOX4*, encoding acyl-CoA oxidases involved in *β*-oxidation steps that contribute to JA formation and *ADH* coding alcohol dehydrogenase participating in the adaptation to adverse conditions [49].

Drought resulted in the downregulation of the *CHLOROPLASTIC OXOENE REDUCTASE* gene (*QORH*) (Fig. 4, Supplementary file 1S7), which encodes an enzyme responsible for detoxifying toxic γ-ketols produced during JAs biosynthesis, downstream of LOX peroxidation [50]. Furthermore, drought both stimulated and decreased the expression of genes encoding allene oxide cyclase (*AOC*) (Fig. 4, Supplementary file 1S7) which catalyzes the stereospecific cyclization of allene oxide to OPDA [51].

**Fig. 5:**
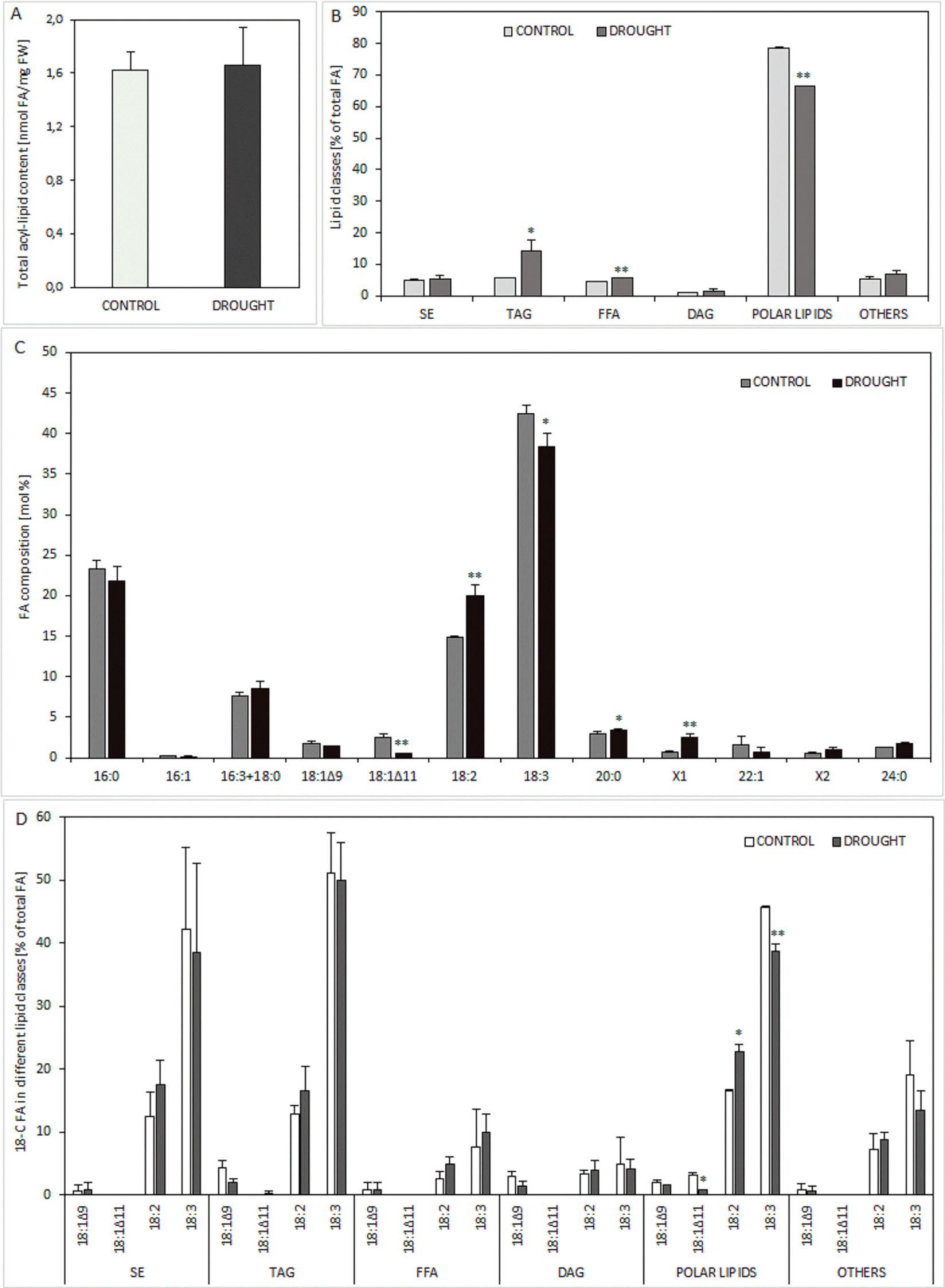
Effect of drought on lipid homeostasis in the roots of yellow lupine. Total fatty acids (FAs) content (**A**), lipid classes (**B**), composition of FAs (**C**), particular 18-C FAs content in various lipid classes (**D**). Control plants were grown in soil maintained at optimal moisture, while stressed plants were subjected to drought conditions. Error bars on the charts represent SD. Significant differences are denoted as ^**^P < 0.01 and ^*^P < 0.05. Abbreviations: SE – sterol esters, TAG – triacylglycerols, FFA – free fatty acids, DAG – diacylglycerol, X1 and X2 – unidentified FAs

Analysis of RNA-seq data revealed changes in drought-induced lipid metabolism, highlighting the stress’s impact on lipid membrane compounds. Consequently, we investigated the drought’s effect on lipid components in lupine roots. While the stressor didn’t alter the total FAs content of lipids (Fig. 5A), it did modify the distribution of lipid classes (Fig. 5B), and influence the composition of FAs (Fig. 5C). Among all FAs, the most abundant in lupine roots were linolenic acid (18:3; up to 45% of all FAs), palmitic acid (16:0; up to 23%), and linoleic acid (18:2; up to 21%) (Fig. 5C). Nevertheless, significant differences between drought-stressed and control roots were observed only for linoleic acid (18:2) and linolenic acid (18:3). The relative amount of the first one was strongly increased under stressor action, but the opposite effect was noted for the second one. A reduction in the relative content of 11-octadecenoic acid (18:1Δ11) was noted during drought conditions. Moreover, lipid analysis of lupine root under stress revealed an accumulation of arachidic acid (20:0) and another very long chain FA (VLC-FA) co-chromatographed with arachidonic acid (20:4) standard and named X1. The content of palmitic acid (16:0), palmitoleic acid (16:1), hexadecatrienoic acid (16:3) along with stearic acid (18:0; both fatty acids co-chromatographed together), oleic acid (18:1Δ9), eruic acid (22:1), fatty acid named as X2 (co-chromatographed with eicosapentaenoic aid standard; 20:5) and, lignoceric acid (24:0) do not differ statistically between control and drouth-treated roots. The observed differences in FAs composition of total lipids prompted further analysis via TLC/GC to investigate specific lipid classes in lupine roots (Fig. 5B) and their FAs composition (Fig. 5D). Five classes of lipids, polar lipids, diacylglycerols (DAG), FFA, TAG, and sterol (SE) and some unidentified lipids categorized as “others” were detected (Fig. 5B). Polar lipids were the most dominant class (approx. 70%), while DAG constituted the smallest fraction (about 2%). Drought stress affected the relative content of 3 identified lipid classes: TAG increased (from about 6% to about 14%), FFA also increased (from about 4 to about 6%), and polar lipids decreased (from about 79% to about 66%). In contrast, statistically significant differences between control and drought-treated roots were not observed for SE, DAG, and lipids treated as one class termed “others”. Analysis of FAs composition across various separated lipid classes indicated that the modulation of 18-carbon FAs by drought was consistent across all lipid classes except for FFA, where the relative amount of 18:3 was increased. Notably, statistically significant differences were observed only in the composition of polar lipids (Fig. 5D).

**Fig. 6:**
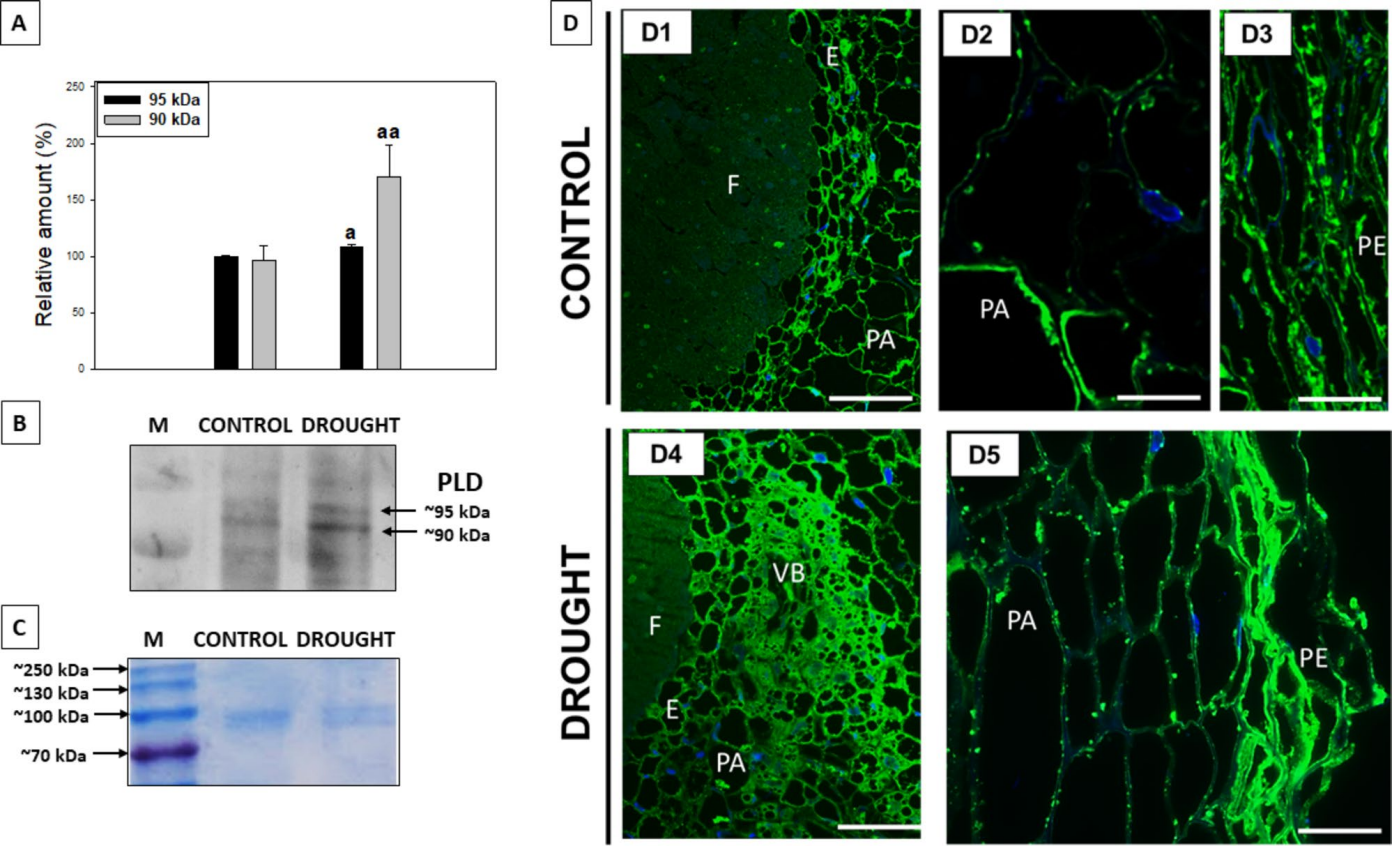
Western blot analysis and immunocytological localization of PLD in the lupine root and nodules under drought conditions. Material was collected from plants subjected to either drought stress or control conditions. Western blot analysis revealed the presence of two bands of size ~ 90 kDa and ~ 95 kDa (marked by arrows, **B**; Supplementary file 2). Densitometric analysis (**A**) using ImageJ software indicated significant differences in band intensities between stressed roots and control samples (100% was set for the isoform in the control variant). ^a^*P*<0.05 and ^aa^*P*<0.01. Coomassie Brilliant blue staining was used for total protein visualization (**C**). Immunostaining of PLD was performed for control (D1-D3) and drought-treated (D4-D5) plants. Green labeling corresponds to the presence of PLD, whereas blue staining indicates nuclei stained with DAPI. Abbreviations: F - fixation zone; E - endodermis cells; PA - nodule parenchyma; PE - periderm; VB - vascular bundle. Scale bar, 100 μm (D1, D4), 50 μm (D2, D3, D5)

The influence of drought on the level and tissue-specific localization of PLD, which hydrolyzes phospholipids components of membranes, was investigated [52]. In lupine roots, two isoforms of PLD were detected, one ~ 95 kDa and the other ~ 90 kDa in size (Fig. 6B). Both of them were strongly accumulated in plants cultivated under drought when compared to the well-watered lupines (Fig. 6A, B). To precisely localize PLD we conducted immunocytochemical reactions using the same antibodies as in Western blot. The enzyme presented in nodules of both control and stressed plants (Fig. 6D). However, in drought-treated lupines, PLD occupied mostly periderm and areas surrounding the vascular bundles near the fixation zone of the nodules (Fig. 6D4, D5). At higher magnification, PLD was observed in the peripheral cytosolic region of cells in the endodermis, nodule parenchyma, and periderm (Fig. 6D).

**Fig. 7:**
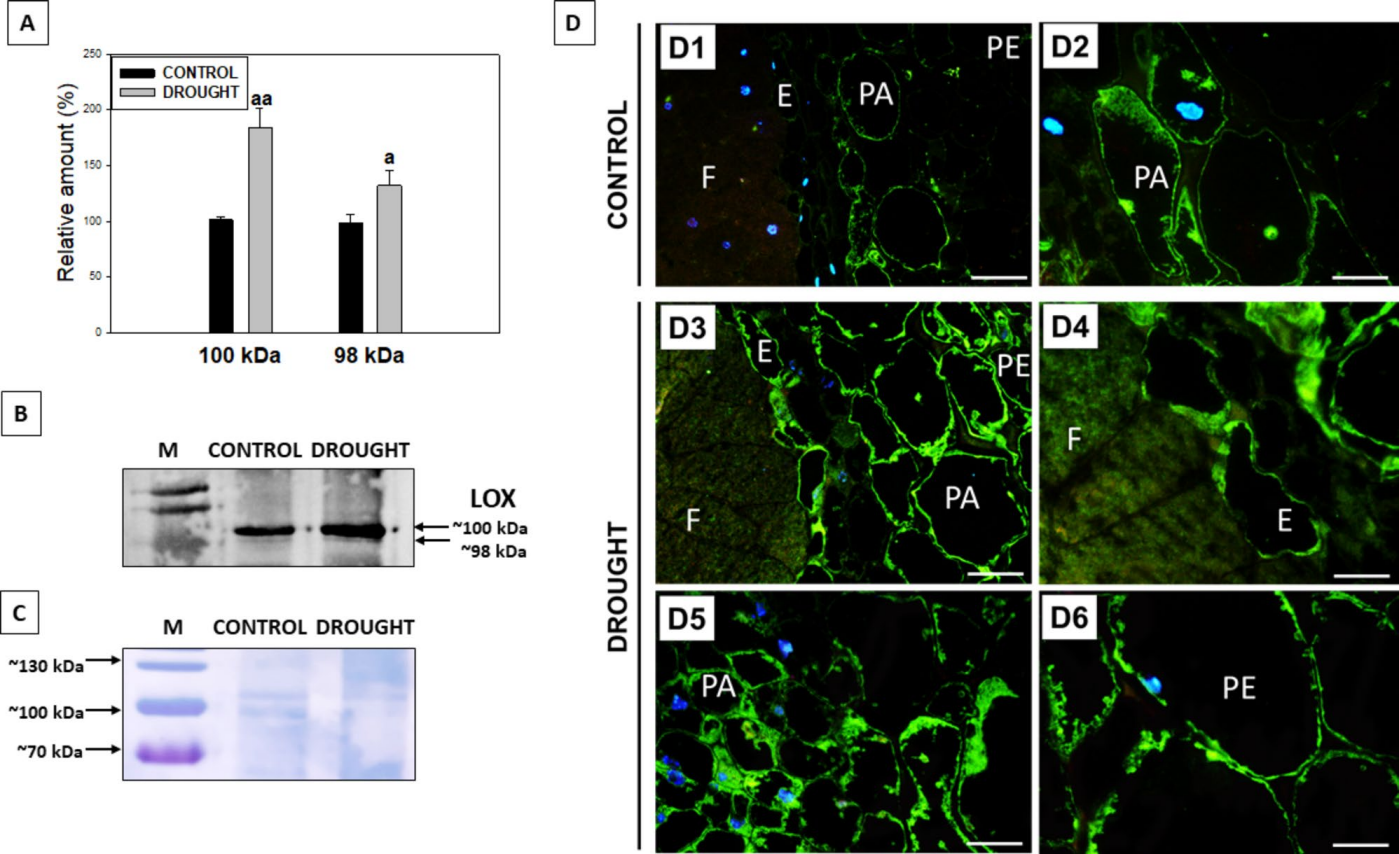
Effect of drought on LOX level (**A**-**B**) and localization (**D**) in lupine root and nodules. Material was collected from plants subjected to drought stress or control conditions. Western blot analysis revealed 2 isoforms of LOX ~ 98 kDa and ~ 100 kDa (indicated by arrows, B; Supplementary file 2). Densitometry analysis of the blots (**A**) using ImageJ software showed significant differences in LOX isoform levels between stressed roots and control (100% was set for the isoforms in the control variant). ^a^*P*<0.05 and ^aa^*P*<0.01. Coomassie Brilliant blue staining was used to visualize total proteins (**D**). Immunolocalization of LOX was made for control (D1-D2) and drought-treated (D3-D6) plants. A green signal indicates LOX presence, while a blue color corresponds to DAPI staining (nuclei). Abbreviations: F - fixation zone; E - endodermis cells; PA - nodule parenchyma; PE - periderm. Scale bar, 50 μm

Modification of membrane lipid composition is linked to changes in the activity of enzymes involved in their metabolism, including LOX, which plays a critical role in regulating lipid bilayer permeability and fluidity [53]. In light of this, the impact of drought stress on LOX abundance and distribution in the roots and nodules of *L. luteus* (Fig. 7) was investigated. Using the Western blot technique, we detected 2 isoforms of LOX of mass ~ 98 kDa and ~ 100 kDa (Fig. 7B). In drought-stressed roots the ~ 98 kDa isoform showed increased accumulation compared to control, while the ~ 100 kDa isoform exhibited a 2 times higher level under stress conditions (Fig. 7A). The same antibody was employed to localize LOX in nodule tissues under drought (Fig. 7D). Immunostaining confirmed that this protein was strongly abundant in the stressed nodules (Fig. 7D1, D2). A significant difference in the presence of LOX was observed in the fixation zone between control and drought conditions (Fig. 7D1, D4). In the control section, fluorescent labeling in the fixation zone and periderm was minimal (Fig. 7D1), with LOX predominantly localized in the cytosol and peripheral regions of nodule parenchyma cells (Fig. 7D2). On the contrary, all analyzed tissues in stressed nodules exhibited strong labeling after treatment with anti-LOX Ab (Fig. 7D3-D6). At the cellular level, LOX was detected in the cytosolic regions across various tissues including parenchyma (Fig. 7D3, D5), endodermis (Fig. 7D3, D4), and periderm cells (Fig. 7D3, D6).

**Fig. 8:**
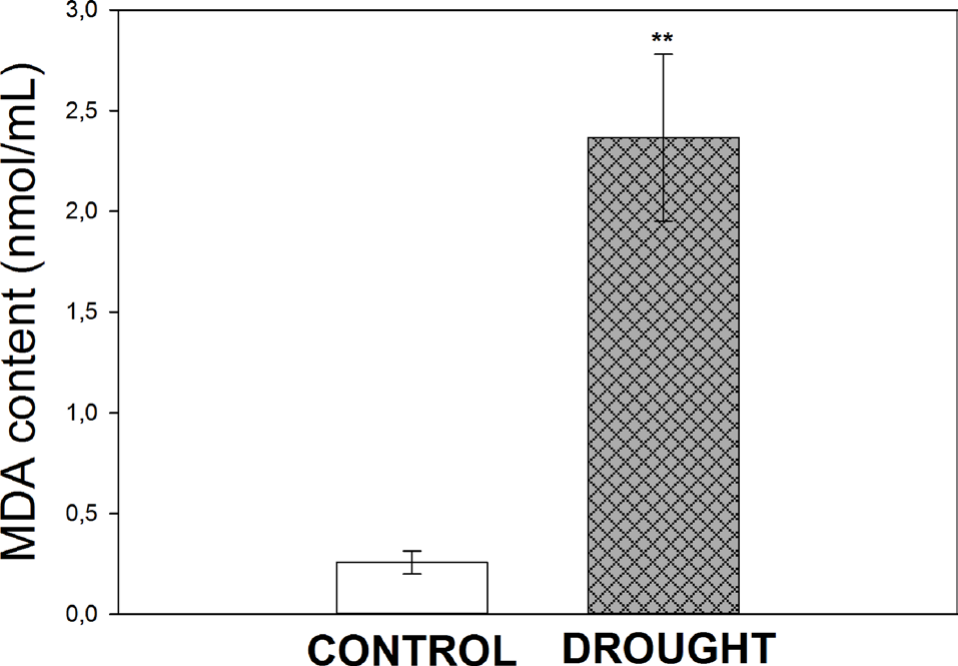
Changes in malondialdehyde (MDA) content in the roots of lupine exposed to drought. Control plants were cultivated under soil of optimal moisture, while stressed plants were exposed to drought conditions. Bars indicate the standard error of the mean (*n* = 3). Significant differences between stressed and control plants are indicated by ^**^P < 0.01

Transcriptomic and biochemical analyses indicating altered lipid homeostasis, our subsequent investigations aimed to assess whether drought induces degradation of cellular membrane lipid components. It has been shown that stress caused the accumulation of MDA (Fig. 8), an indicator of membrane lipid oxidation [54]. Its level was ~ 9 times higher under drought conditions compared to the control.

### Effect of drought on the redox homeostasis in lupine root

Accumulated ROS can mediate the oxidative degradation of lipids, essential structural components of membranes crucial for maintaining cell stability [55]. ROS can also influence cell physiology through protein carbonylation and affect gene expression by interacting with nucleic acids and transcription factors [56].

Transcriptomic analysis indicated a diverse gene expression profile related to oxidative stress (Supplementary file 1S8). Under drought conditions, there was a notable upregulation of genes coding the elements of enzymatic antioxidant system, including *SOD*, *CAT*, and *GLUTATHIONE PEROXIDASE* (*GPX*), several peroxidases i.e. *HEME PEROXIDASE 2* (*HPX2*), manganese peroxidase 2 (*PEM2*), and also *PER1*, *PER9*, *PER42*, *PER47*, *PER51*. In contrast, the expression of *APX* gene and other peroxidases (*PER4*, *PER5*, *PER6*, *PER10*, *PER12*, *PER17*, *PER21*, *PER38*, *PER53*, *PER64*, *PER66*, *PER72*, and *PERE5*) showed a significant downregulation (Supplementary file 1S8). Furthermore, genes encoding respiratory burst oxidase homologs (*RBOHB*, *RBOHC*, and *RBOHE*) were downregulated in the lupine roots.

The analysis identified five isoforms of SOD, a key enzyme in the first line of defense against ROS-mediated stress in the *L. luteus* roots (Table 1). Their occurrence in lupine or other legumes is associated with different cellular compartments, such as chloroplasts/cytosol (Fe-SOD), mitochondria (Mn-SOD1, Mn-SOD2), and cytosol (Cu/Zn-SOD1, Cu/Zn-SOD2) [57, 58]. In the roots of *L. luteus* under control conditions, Cu-ZnSOD2 exhibited the lowest activity, while Cu-ZnSOD1 showed the highest activity (Table 1). The activity of all SODs isoforms in *L. luteus* was influenced by drought. Despite Cu/Zn-SOD1 having the highest activity (~ 23,08 U mg^*−* 1^ protein) among all isoforms (Table 1), Cu/Zn-SOD2 showed the most significant increase in activity due to drought. By contrast, Mn-SOD1 was negatively regulated by the stress factor.

**Table 1:** Drought-dependent activity of superoxide dismutase (SOD) in lupine roots and nodules. The SOD isoenzyme profile was analyzed in roots with nodules collected from plants grown under drought conditions and from control lupines cultivated in soil with optimal moisture. Data are presented as means ± SE. Significant differences between drought-stressed and control plants are indicated by ^aa^*P*<0.01, ^a^*P*<0.05 (Student’s t-test)

SOD activity (U mg^-1^ protein)	Control	Drought
Fe-SOD	5.44 ± 0.03	13.26 ± 0.09 ^aa^
Mn-SOD1	2.90 ± 0.02	2.20 ± 0.01 ^a^
Mn-SOD2	2.74 ± 0.05	3.74 ± 0.03 ^a^
Cu/Zn-SOD1	7.80 ± 0.06	23.08 ± 0.15 ^aa^
Cu/Zn-SOD2	1.80 ± 0.02	7.86 ± 0.06 ^aa^

To elucidate the complex ROS-dependent response of roots to drought, the level of H_2_O_2_ and the activity of two detoxifying enzymes, CAT and APX (Table 2), were also determined. Upon stress, the H_2_O_2_ content increased significantly, reaching levels ~ 4 times higher than those in the control. It was accompanied by elevated CAT activity, which reached ~ 1142 µM/min g FW, more than 5 times higher than in the control. The antioxidant defense response was also reflected by the stimulation of APX activity. However, this effect was not such strong as one for the CAT; the APX activity in stressed roots was about ~ 9% higher than in the control.

**Table 2:** Drought stress influences the redox status in lupine roots and nodules. Analyses of hydrogen peroxide (H_2_O_2_) content and activity of catalase (CAT) and ascorbate peroxidase (APX) were performed in roots and nodules from control plants grown optimally moist soil and from drought-stressed lupines. Data are presented as means ± SE. Significant differences between drought-stressed and control plants are indicated by ^aa^*p*<0.01 and ^a^*p*<0.05 (Student’s t-test)

Parameter	Control	Drought
H_2_O_2_ content (µM g^-1^ FW)	400.00 ± 25.42	1758.00 ± 75.25 ^aa^
CAT activity (U mg^-1^ protein)	210.00 ± 15.67	1142.02 ± 34.75 ^aa^
APX activity (U mg^-1^ protein)	33.65 ± 2.66	36.71 ± 0.49 ^a^

## Discussion

Drought strongly affects the yield of yellow lupine [7]. This study identifies extensive gene expression variations in drought-stressed lupine roots compared to controls, supported by numerous DEGs (Fig. 1A). Protein functions suggest involvement in both early and late plant responses to drought stress (Supplementary file S6). Early-response genes include transcription factors, kinases, phosphatases, proteins of phospholipid metabolism, and components of signal transduction pathways. The second group comprises genes encoding proteins responsive to dehydration or ABA, contributing to water and ion transport, cellular structure stability, ROS protection, and osmoprotectant synthesis.

To adapt to drought, lupine roots undergo a series of changes mediated by phytohormones, crucial elements induced by environmental stresses. Our research highlights the importance of phytohormones like auxins, CKs, GAs, ABA, ET, BRs, SA, and JAs in drought responses, supported by the highest number of DEGs annotated to hormone signaling pathways (Fig. 1C). Auxins act via ARF transcription factors, regulating osmoprotectant content and enhance root growth under stress [59]. Drought stress negatively impacts genes involved in auxin biosynthesis and signaling, including *LAX*, *TIR*, *AUX*/*IAA*, and *ARF* (Fig. 2), potentially reducing root growth [60]. However, certain abiotic factors induce *ARF* expression as observed in tomato and *G. max* [61]. In lupine roots, differential regulation of *SAUR* genes (Fig. 2), known for promoting drought tolerance [62], reflects their diverse roles in stress responses. A similar pattern was noted for CKs-related genes (Fig. 2). While CKs can have negative effects on drought-treated plants, they also mitigate premature senescence under stress conditions [63]. Previous studies highlight drought’s impact on *AHP4* expression in *A. thaliana* [64]. The downregulation of CK responses may enhance plant survival, supported by research on transgenic plants [65]. Differential regulation of GAs receptor genes (*GID1B* and *GID1c*) and repressor genes (*SCL*, *DELLA*) in lupine underscores the complex response to drought (Fig. 2), activating or inhibiting specific components of the GAs signaling pathway. In *Camellia sinensis*, both drought and cold stress affect *GID1C* expression [66].

Diverse expression patterns of ABA receptors under abiotic stresses in leaves and roots have been documented in *Z. mays* [67]. In this study, the down-regulation of ABA receptors (Fig. 2) may affect signal perception efficiency, potentially disrupting the initial step of the core signaling pathway. However, the impact is nuanced by differential regulation of various *SnRK2* kinases (Fig. 2), crucial for ABA transduction pathway.

Lupine responds to drought by enhancing ET perception, as evidenced by the upregulation of *ETR2* expression (Fig. 2). Conversely, the accumulation of *EBF1* transcripts suggests negative regulation of the ET-signaling pathway. Other transcription factors may also participate in the ET-mediated drought responses, considering the increased hormone synthesis observed in lupine roots under stress action [4]. Downregulation of genes involved in early steps of BRs signaling suggests a limited role for these hormones in drought responses (Fig. 2). These findings are consistent with reports of improved drought tolerance when BRs signaling is disrupted [68]. However, BRs have been shown to play a beneficial role in stress-evoked mechanisms in *A. thaliana*, wheat, and *Brassica* [69], which aligns with our findings of upregulated BR-responsive genes in drought-treated roots (Fig. 2).

Plants enhance abiotic stress tolerance through pathways involving SA [70]. In our experiment, however, most SA signaling components in the roots are downregulated by drought (Fig. 2). This may be explained by SA primarily exerting its effects under stress in above-ground plant parts, where it improves drought resistance by protecting antioxidant elements and maintaining photosynthetic efficiency [71].

The JAs-mediated response to drought involves accelerated biosynthesis and catabolism [72]. Transcriptional regulation of JAs catabolism varies in lupine roots under drought, suggesting a role for GH3 in JA-Ile formation during stress (Fig. 2). Despite the downregulation of *COI1* other JAs derivatives such as MeJA observed in *Oryza sativa*, *G. max*, and *Brassica oleracea* may still be involved [73–75]. The induction of JAs perception is supported by the downregulation of most genes encoding JAZ repressor proteins (Fig. 2).

Transcriptional regulation of lipid metabolism is a mechanism for lupine roots to encounter drought (Fig. 3). Upregulation of *KASC1* suggests enhanced biosynthesis of FAs containing more than 16 C atoms. However, the relative proportions of 16 C FAs (sum of 16:0, 16:1, and 16:3) in lipid extracts from control and drought-treated roots were comparable (Fig. 5C). In stressed roots, accumulation of 20:0 and FAs referred to as X1 (Fig. 5B) likely results from coordinated action of KAS II (a product of *KASC1*) and the cytosolic FAs elongation system of which 3-ketoacyl-CoA synthase (KCS) encoding gene was upregulated (Fig. 3). Downregulation of genes encoding cytosolic and plastid acetyl-CoA carboxylases (ACCases), crucial for *de novo* synthesis of FAs, along with downregulated genes in the FAs biosynthetic pathway in drought-exposed roots (Fig. 3), suggests reduced net intensity of FAs biosynthesis compared to control roots. Nevertheless, total FAs content in acyl-lipids of drought-treated and control roots was similar (Fig. 5A). Despite this, root growth and function under drought conditions are typically less vigorous than under optimal conditions [4], suggesting downregulated genes may represent a mechanism adjusting FAs biosynthesis the root development needs. Changes in relative FAs amounts (Fig. 5C) represent real adaptations to drought conditions, potentially improving membrane properties crucial for water-deficient environments. The simultaneous increase in 18:2 and decrease in 18:3 content (Fig. 5C) indicates reduced Δ15 desaturase activity, possibly with unchanged or elevated Δ12 desaturase activity. Transcriptomic data supports this hypothesis, showing upregulation of the gene encoding Δ12 desaturase (Supplementary file S3), however, the activity of both desaturases was not assessed. Our findings align with reports of decreased linolenic acid (18:3) content observed in pea [76] and soybean under drought [77]. Changes in 18-carbon FAs level due to drought may disrupt membrane fluidity, stability, and integrity, as they are major components of the lipid bilayer. Generally, TAG level remains unchanged in vegetative tissues; but can increase under stressors like water deficit [78]. Environmental stresses are linked to the accumulation of toxic intermediates such as FFA, which can trigger programmed cell death, while TAGs may sequester these intermediates, protecting cells [79]. This might explain the simultaneous rise in both FFA and TAGs observed in drought-stressed lupine roots (Fig. 5B).

Drought negatively affects *PLA2* and *LOXs* expression in lupine roots (Figs. 3 and 4), likely reducing JAs biosynthesis. Similar *PLA2* downregulation occurs in drought-stressed *Arabidopsis* [80]. Conversely, drought increases PLD, which produces phosphatidic acid (PA) – a sensor of external factors [81] – in lupine roots and nodules (Fig. 6). PLD*α*1 and PA play multifaceted roles in plant-pathogen interactions and nodulation in *G. max* [82–84]. PA participates in lipid degradation, turnover, and signaling of ABA and ET [83, 85, 86], crucial for nodule response to drought stress in lupine [4] (Fig. 2). Two PLD isoforms observed in the nodules under optimal moisture conditions (Fig. 6) may be necessary for proper nodule morphogenesis. Increased PLD isoforms under drought (Fig. 6) suggests their involvement in stress responses and hydrolysis of membrane lipids, aligning with findings in *A. thaliana* [87], *Citrus sinensis* [88], *Vigna radiata* [89]. PLD is present in both soluble fractions and membranes [86, 87]. The observed PLD localization pattern in the dehydrated lupine nodules (Fig. 6) indicates its role in membrane component hydrolyzing and disorganization via phospholipid catabolism. PLD presence in the periderm and endodermis (Fig. 6) suggests drought severely damages nodule cell membranes, which act as oxygen diffusion barriers. This disruption, through FAs hydrolysis, activates LOX-mediated linoleate oxidation, leading to the production of 12-oxo-phytodienoic acid (12-OPDA) and phytohormones like JAs [90].

A strong LOX burst in the lupine root nodule zone indicates its involvement in drought response, with one isoform more sensitive to stress (Fig. 7). These results are consistent with our previous findings showing soil water deficit increases LOX in lupine flower abscission zone [6]. A ~ 95 kDa LOX isoform has also been observed in roots and nodules of *Medicago truncatula*, *Crasualina glauca*, and *Datisca glomerata* [91]. In our experiment, LOX is mainly localized in tissues around the fixation zone of well-watered lupine roots, similar to other legumes where it is absent from nitrogen-fixing cells [91]. This parenchyma-specific LOX presence aligns with findings in pea [92]. Drought has increased LOX activity in *Olea europea* leaves and roots [93], suggesting that LOX in lupine roots nodules (Fig. 7) may also be more active under drought, despite gene downregulation (Fig. 4). Free sterols maintain plasma membrane integrity, permeability, and fluidity [94, 95]. Their esterification with FAs excludes them from the membrane. Thus, the reduced SE in drought-exposed lupine roots (Fig. 5C) may indicate increased free sterols. However, drought effect on free sterol content was not assessed in this study.

TAG accumulation in drought-treated lupine roots (Fig. 5C) may be a defense reaction against stress by serving as an energy source for membrane recovery [96]. TAG formation also reduces FFA (Fig. 5C). Under stress, converting FFAs into TAGs protects against ROS damage [67]. TAG biosynthesis can be also stimulated by ABA, a main sensor of water deficit responses in plants [97].

Soil water deficit disrupts the redox balance in lupine [7], but no transcriptomic analyses have been performed on drought-stressed lupine tissues. This study shows water deficit induces redox-related genes for H_2_O_2_ detoxification (e.g. *SOD*, *CAT*), confirmed by biochemical measurements (Tables 1 and 2). Stress induces SOD activity in pea [98] and wheat [99–101], while in sunflower [102], and *Aegilops squarrosa* L [103] negative impact has been observed, and no effect in maize [104].

Various isoforms (cytosolic Cu/ZnSODs and chloroplast/cytosol FeSOD) help defend lupine roots against oxidative stress under drought (Table 1). A similar response of Cu/ZnSOD, FeSOD, and MnSOD in the leaves of *Lupinus angustifolius* [105] indicates ROS overproduction and induction of scavenging mechanism. Increased H_2_O_2_ level in roots correlates with higher CAT activity (Table 2), suggesting redox imbalance. Drought has less effect on APX activity (Table 2), likely because APX is more active in photosynthetic tissues [106, 107]. The dominant role of CAT in H_2_O_2_ detoxification in roots has been also demonstrated in cotton [108] and *Medicago* under water stress [109]. ROS act as regulatory signals for lipid peroxidation, generating *α*- and *β*-aldehydes like MDA [110]. The accumulation of MDA in drought-stressed lupine roots (Fig. 8) indicates oxidative damage of membrane lipids.

In summary, response of lupine root to drought involves modifications in the homeostasis of hormones, lipids, and redox status. These insights are derived from a comprehensive transcriptional network analysis and detailed biochemical studies. The integration of the presented results suggests a metabolic pathway in which upregulated *Δ12 DESATURASE* gene expression and PLD activity lead to the production of high levels of linoleic acid (18:2), which is then enzymatically oxidized by LOX. This oxidation process results in membrane disintegration and subsequent accumulation of MDA, a marker of oxidative stress, supported by increased activity of SOD, APX, CAT. Additionally, the conversion of FFAs into TAGs provides another mechanism for protection against ROS-induced damage. Our findings offer novel molecular insights essential for selecting and developing drought-resistant lupine cultivars, laying the groundwork for future advancements in stress tolerance.

## Electronic Supplementary Material

Below is the link to the electronic supplementary material.

Supplementary Material 1

Supplementary Material 2

## Data Availability

Sequence data that support the findings of this study have been deposited in The Sequence Read Archive (SRA) available through NCBI servers with the primary accession code PRJNA890161 https://www.ncbi.nlm.nih.gov/sra/?term=PRJNA890161 All of the rest data generated and analyzed during this study are included in this published article and its supplementary files.
