# Supplementary material for: Exploring the response of yellow lupine (Lupinus luteus L.) root to drought mediated by pathways related to phytohormones, lipid, and redox homeostasis
Source: BMC Plant Biol. 2024 Nov 6;24:1049. doi: 10.1186/s12870-024-05748-4 (PMC11539565; doi:10.1186/s12870-024-05748-4)
Supplement: Supplementary file 2 — Supplementary Material 2 [file 12870_2024_5748_MOESM2_ESM.pptx]

## Slide 1
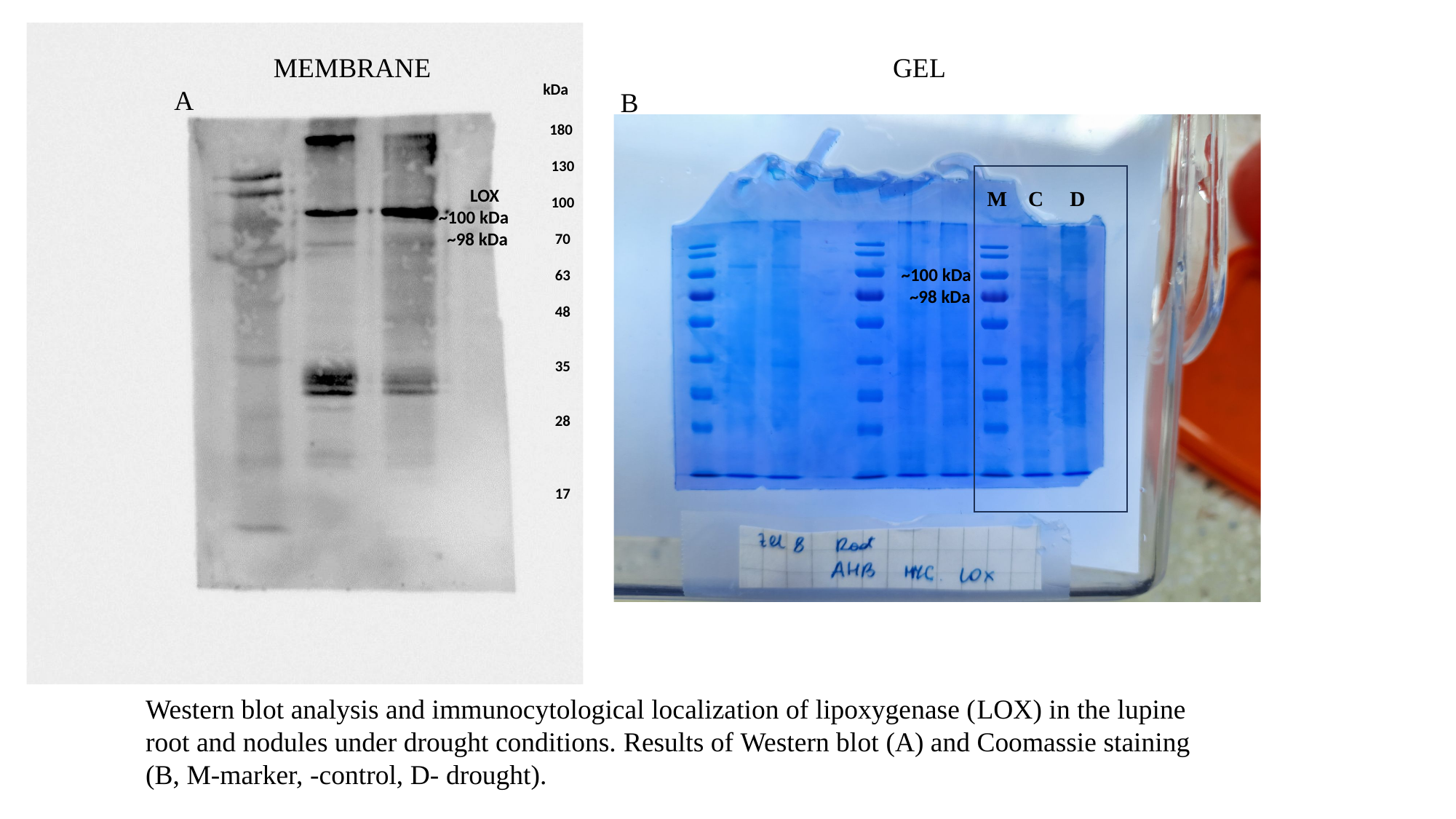

MEMBRANE
GEL
kDa
A
B
180
130
100
70
63
48
35
28
17
 LOX
~100 kDa
 ~98 kDa
M C D
~100 kDa
 ~98 kDa
Western blot analysis and immunocytological localization of lipoxygenase (LOX) in the lupine root and nodules under drought conditions. Results of Western blot (A) and Coomassie staining (B, M-marker, -control, D- drought).

## Slide 2
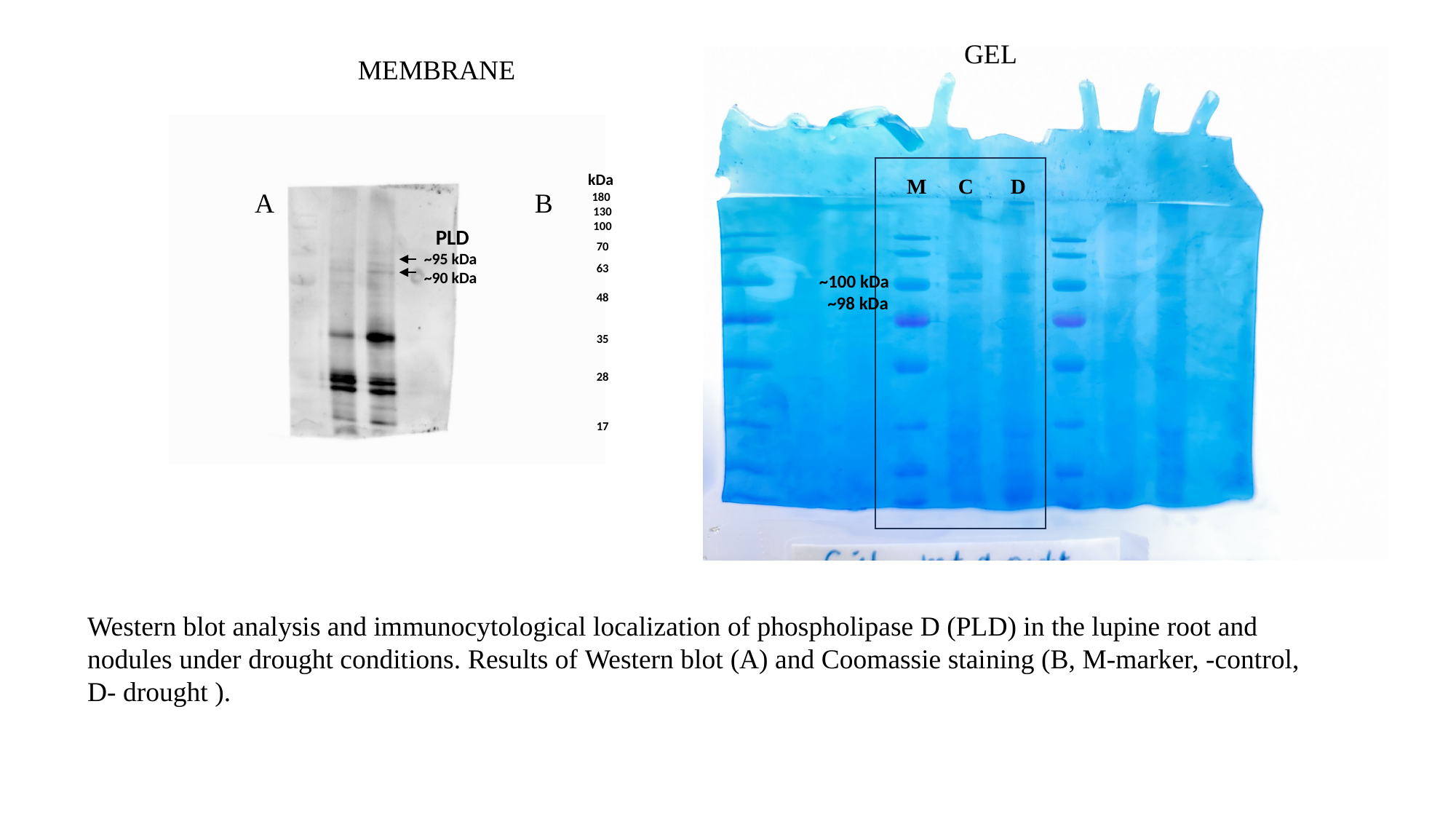

GEL
MEMBRANE
kDa
M C D
A
B
180
130
100
70
63
48
35
28
17
 PLD
~95 kDa
~90 kDa
~100 kDa
 ~98 kDa
Western blot analysis and immunocytological localization of phospholipase D (PLD) in the lupine root and nodules under drought conditions. Results of Western blot (A) and Coomassie staining (B, M-marker, -control, D- drought ).
